# Functional Cross-Talking between Differentially Expressed and Alternatively Spliced Genes in Human Liver Cancer Cells Treated with Berberine

**DOI:** 10.1371/journal.pone.0143742

**Published:** 2015-11-25

**Authors:** Zhen Sheng, Yi Sun, Ruixin Zhu, Na Jiao, Kailin Tang, Zhiwei Cao, Chao Ma

**Affiliations:** 1 School of Life Science and Technology, Tongji University, Shanghai, People’s Republic of China; 2 Key Lab of Systems Biology, Shanghai Institutes for Biological Science, Chinese Academy of Science, Shanghai, People’s Republic of China; International Centre for Genetic Engineering and Biotechnology, ITALY

## Abstract

Berberine has been identified with anti-proliferative effects on various cancer cells. Many researchers have been trying to elucidate the anti-cancer mechanisms of berberine based on differentially expressed genes. However, differentially alternative splicing genes induced by berberine might also contribute to its pharmacological actions and have not been reported yet. Moreover, the potential functional cross-talking between the two sets of genes deserves further exploration. In this study, RNA-seq technology was used to detect the differentially expressed genes and differentially alternative spliced genes in BEL-7402 cancer cells induced by berberine. Functional enrichment analysis indicated that these genes were mainly enriched in the p53 and cell cycle signalling pathway. In addition, it was statistically proven that the two sets of genes were locally co-enriched along chromosomes, closely connected to each other based on protein-protein interaction and functionally similar on Gene Ontology tree. These results suggested that the two sets of genes regulated by berberine might be functionally cross-talked and jointly contribute to its cell cycle arresting effect. It has provided new clues for further researches on the pharmacological mechanisms of berberine as well as the other botanical drugs.

## Introduction

Alternative splicing is a tightly regulated process during gene expression that can produce different forms of a protein from the same gene. Moreover, it has been proved that alternative splicing can also determine binding properties, intracellular localization, enzymatic activity, protein stability and posttranslational modifications of a large number of proteins [1]. In recent years, more and more pharmacological researchers have found that small molecular drugs could exert their pharmacological effects not only through regulating transcription levels of genes but also through changing gene alternative splicing [2].

Berberine, an isoquinoline quaternary alkaloid isolated from Berberis species [3], has a wide spectrum of pharmacological effects such as anti-microbe, anti-diabetes and anti-inflammation [4]. Clinically, it has been used to treat a range of disorders, including coronary artery disease, diabetes, non-alcoholic fatty liver disease, hyperlipidaemia, metabolic syndrome, obesity and polycystic ovary syndrome (https://www.clinicaltrials.gov/). Recently, accumulating studies have found that berberine also possessed potent anticancer activity, with few or minimal undesired toxic effects [5, 6]. Therefore, many researchers have been trying to elucidate the anti-cancer mechanisms of berberine based on gene differential expression. For instance, it is reported that berberine could induce apoptosis in HepG2 cells through AMPK-mediated mitochondrial pathway by increasing the ratio of Bax/Bcl-2 [7]. In our previous study, we also have found that berberine could induce G1 cell cycle arrest in BEL-7402 cells partially via disturbing the interaction of calmodulin with CaMKII and blocking subsequent p27 protein degradation. [8]

Although these works have provided some fundamental knowledge about the anticancer mechanism of berberine, alternative splicing in cancer cells after treated with berberine has not been reported yet. Firstly, it has been implied that many anticancer drugs could inhibit the growth of cancer cells by altering gene alternative splicing [9–11]. In addition, it is reported that gene transcription and alternative splicing might be physically and functionally coupled [12, 13], thereby jointly contribute to the pharmacological actions of these drugs. Therefore, it is necessary to simultaneously measure the differentially expressed genes (DEGs) and the differentially alternatively spliced genes (DASGs) in cancer cells after treatment with berberine and systematically examine whether there is any kind of functional cross-talking between these two sets of genes.

For the question mentioned above, the latest next-generation sequencing technology (RNA-seq) was used to obtain the transcriptomic profiling of BEL-7402 cells after berberine treatment. Then, the DEGs and DASGs induced by berberine were carefully detected by a suite of sequence analysis tools. Finally, the functional crosstalk between these DEGs and DASGs was statistically analysed and their possible contributions to the anticancer effect of berberine were discussed.

## Materials and Methods

### Cell culture

The established human liver cancer cell line (BEL-7402, Cat. No.: TCHu_10) [8, 14, 15] was bought from and deposited in the cell bank of the institute of Biochemistry and Cell Biology, Shanghai Institutes for Biological Sciences, Chinese Academy of Science (http://www.cellbank.org.cn/) and kindly provided by Dr. Xuan Liu (Shanghai Institute of Materia Medica, Chinese Academy of Sciences, Shanghai, P.R.China). Cells were maintained in a humidified 37°C atmosphere containing 5% CO_2_ and cultured in RPMI-1640 medium (GIBCO, Grand Island, NY, USA) supplemented with 10% fetal bovine serum, 2 mM/L-glutamine, 50 units/mL penicillin, and 50 mg/mL streptomycin. In this study, we have pooled 3~5 biological repeats into one RNA-seq experiment to achieve the same amount of total RNA content. More specifically, at the cell culturing section, cancer cells in 8~10 different petri dishes were divided into two equal groups. One group was used as berberine treated group, the other was used as control/untreated group.

### RNA isolation

For extraction of total RNA, cells were plated on 10-cm plates (Corning, Acton, MA, USA) in complete medium for 12 h. Then, 50 μM berberine (purity ≥ 98%, Tauto Biotech, Shanghai, China, IC50 according to Ref.[8]) was added and left in contact for 24 h. After that, cells were rinsed in PBS and lysed in TRIzol reagent (Invitrogen, Carlsbad, CA). Residual contaminating genomic DNA was removed from the total RNA fraction using Turbo DNA-free kit (Ambion, Austin, TX, USA). mRNA was isolated from DNA-free total RNA using the Dynabeads mRNA Purification Kit (Invitrogen, Carlsbad, CA, USA) according to the manufacturer’s protocol. Finally, to obtain enough quantity of mRNA content for sequencing, the total RNA samples from each group were pooled together as one pooled sample.

### Transcriptome sequencing

mRNA selection, library preparation and sequencing was performed by Shanghai Ceneter for Bioinformation Technology (SCBIT) on an Illumina Hiseq 2000 sequencing platform according to manufacturer specifications. Briefly, mRNA was selected using oligo (dT) probes and then fragmented using divalent cations. cDNAs were synthesized using random primers, modified and enriched for attachment to the Illumina flow cell. We sequenced two 60-cycle 100 bp × 2 paired-end lanes, generating ~83.1 million reads.

### Read alignment

Raw sequencing reads were trimmed out Ns and low quality regions (average base quality score in a 5-mer slide-window less than 20, that is, sequencing error rate ≤1%). Read-pairs with at least 36 bp left in each read were kept as clean reads. Sequencing quality was assessed using FastQC [16]. Then, clean reads were mapped to human reference genome (Ensembl GRCh37.66 = hg19) using aligner Tophat [17] (version 2.0.6) with parameters (-r/—inner-mate-distance: 70, -g/—max-multihits: 1,—no-coverage-search,—no-novel-junc, the others as default). The outputs of Tophat were used as the inputs for HTSeq [18]and AltAnalyze [19] to perform gene differential expression or alternative splicing analysis respectively.

### Differential gene expression analysis

DEGs (Differentially Expressed Genes) refer to those genes with significant changes in their expression level. Firstly, the number of reads mapped to the genomic region of each gene for each sample was estimated with HTSeq [18]. Then DESeq [20] was used to assess the difference in gene expression between different samples. Finally, DEGs were filtered by the following rules: 1) the absolute value of log2 [fold-change] was greater than 1. 2) The p-value was less than 0.05.

### Differentially gene alternative splicing analysis

**Fig 1:**
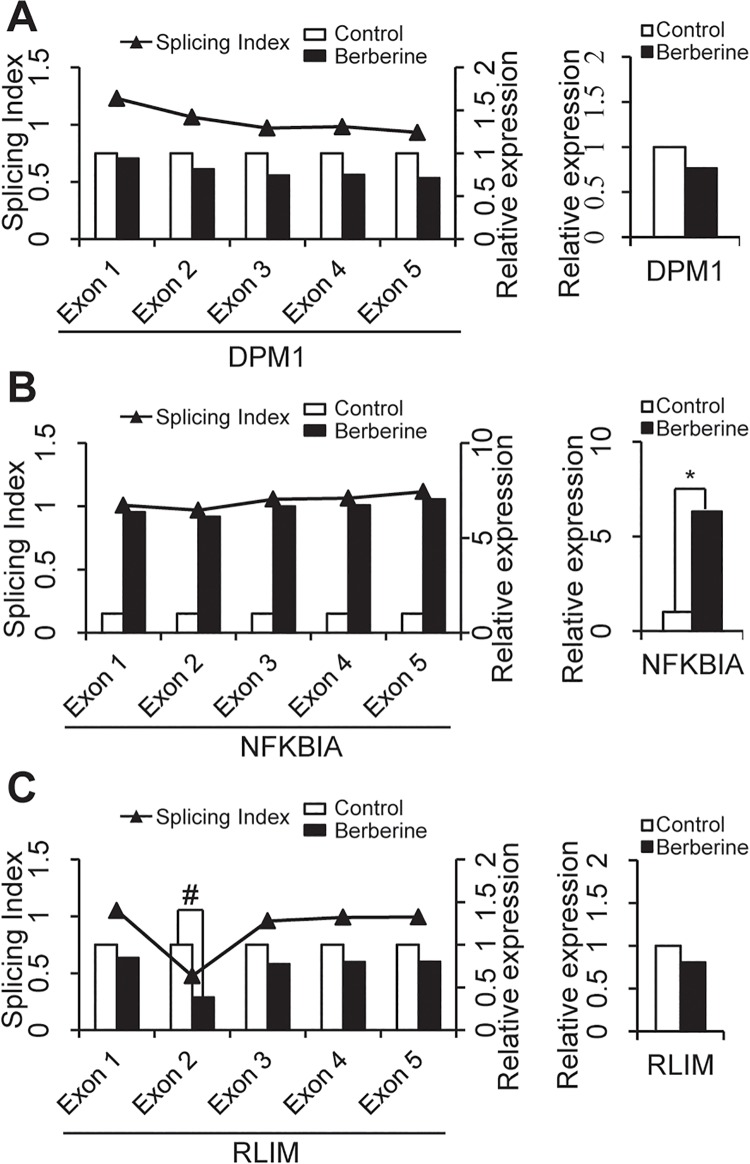
Representation of differentially expressed and alternatively spliced genes after berberine treatment. A. The expression or alternative splicing of DPM1 gene remained unchanged after berberine treatment. B. The splicing pattern of NFKBIA gene remained unchanged after berberine treatment as indicated by the splicing index (SI) value of each of its exons. However, the expression of NFKBIA gene was upregulated by more that 2-fold after berberine treatment (p<0.05) since each of its exons were overexpressed. C. The splicing pattern of RLIM gene was altered by berberine treatment as indicated by the SI value of exon 2 (SI < 0.5) within RLIM gene. But the expression of RLIM gene did not changed significantly after berberine treatment. Relative expression, the relative expression level of the gene or all its exons compared to the control sample. ^*^, DEG; ^#^, DSAG.

DASGs (Differentially Alternative Spliced Genes) refer to those genes with no or small variations in their whole-gene expression level but significant changes in their exon usage, especially those alternative exons (Fig 1). Differential splicing analysis describes the differences in alternative splicing site usage between two samples, which is critical for studies involving mechanisms of alternative splicing and its regulation, and able to uncover functional diversity that is missed by differential gene expression analysis [21]. Dozens of available software tools and packages, such as Cuffdiff2 [22], MISO [23], DEXseq [24], DEGseq [25], DiffSplice [26], Splicing compass [27], AltAnalyze [19] and etc., took conceptually different approaches that could identify differential splicing at the level of isoform/transcript, exon, or both. However, the choice of a suitable approach for a study depends on the experimental objective and expected outcome. Among these approaches and packages, only AltAnalyze provides a graphical user interface, while the rest are run on the commend line. Besides, AltAnalyze provides a convenient workflow to extract the significant alternative splicing regulation events and subsequently investigate the potential biological implications of such splicing events and related mechanisms in the context of molecular interactions and binding sites [19]. Multiple algorithms are available in AltAnalyze to identify individual features (exons or junctions) or reciprocal junctions that are differentially regulated relative to gene expression changes. In this work, single feature analysis (Splicing Index, SI) was performed immediately after reciprocal junction analyses (Analysis of Splicing by Isoform Reciprocity, ASPIRE) [28] on the same list of expressed features, which allows us to examine alternative exons predicted by pairs of reciprocal junctions in addition to those predicted by a single regulated exons/junctions (see AltAnalyze-Manual). Splicing index is one measurement of differential splicing between two samples. Exons with SI value larger than 1 means more inclusion of the exon in the matured mRNA during splicing process in the treated sample compared with the control sample, while those with SI value less than 1 means less inclusion but more skipping of the exon, and those with SI value equal to 1 means there is no change in the usage of the exon.

Differentially alternative splicing analysis was carried out using AltAnalyze with default parameters. Firstly, there were three criteria of exons to perform differentially gene alternative splicing analysis: 1) At least 5 reads were mapped to the exon; 2) the RPKM (Reads Per Kilo-base of exon model per Million mapped reads)value of the exon was larger than 0.3; 3) the expression change of the corresponding gene was less than 2-fold. Then, two exon-centric algorithms were used to detect the differentially alternative splicing exons: Splicing Index (SI) and Analysis of Splicing by Isoform Reciprocity (ASPIRE) [28]. The SI value for each exon or junction was calculated as below.

$$SI\left(exon_{i}\right)=\left(\frac{RPKM_{treatment}\left(exon_{i}\right)}{RPKM_{treatment}\left(gene\right)}\right)÷\left(\frac{RPKM_{control}\left(exon_{i}\right)}{RPKM_{control}\left(gene\right)}\right)$$ (1)

In which, *RPKM*(*exon*_*i*_) and *RPKM*(*gene*) were the expression value of the i-th exon and the gene respectively. In SI algorithm, exons with SI>2-fold were called AS-exons_SI. In ASPIRES algorithm, exons with ΔI>0.2 were called AS-exons_ASPIRE. The details of these two algorithms have been described in AltAnalyze workflow (http://www.altanalyze.org/). Only overlapped AS-exons in both algorithms were chosen for further functional analysis. And the genes containing these exons were called differentially alternative spliced genes (DASG).

### Gene Set Functional Enrichment Analysis

Gene set enrichment analyses were performed for the functional annotation of the DEGs and DASGs separately. Functional Annotation Tools in DAVID Bioinformatics Resources [29] were used to carry out these analyses. Those gene ontology (GO) [30] biological process terms or Kyoto Encyclopedia of Genes and Genomes (KEGG) [31] pathways with enrichment p-value less than 0.05 (modified Fisher’s exact test) and more than two genes were considered as significantly enriched functions for further analysis.

### Local co-enrichment analysis along chromosome sequences

**Fig 2:**
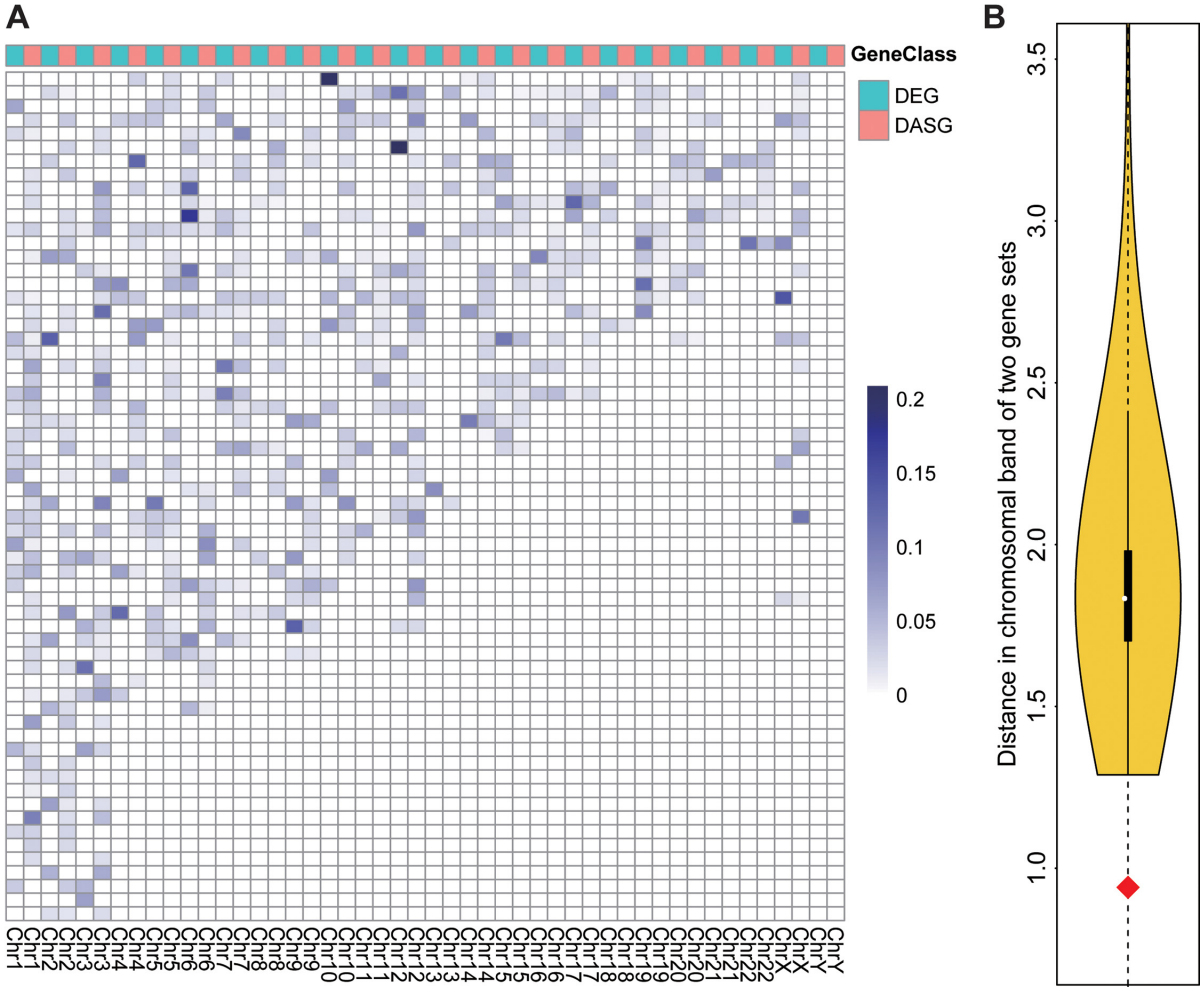
Positional co-enrichment of DEGs and DASGs along 24 chromosomes. A. Global intensity plot of positional enrichment for DEGs and DASGs. Each cell represents a chromosomal band and the intensity of its color is proportional to the corresponding enrichment of DEGs or DASGs in this band, which is normalized by both the total amount of genes in the band and the number of either DEGs or DASGs. Each column represents the chromosome containing all the bands and the color, either blue or red, ahead of the column distinguishing the class of genes from DEGs and DASGs. B. The violin plot for the positional co-enrichment score (distance) between two gene sets. The curve covering the yellow region represents the distance distribution of two random gene sets in the same size of DEGs and DASGs respectively. A shorter distance means a higher co-enrichment of these two gene sets. The red diamond point refers to the distance between DEGs and DASGs.

To test whether the DEGs and DASGs showed similar enrichment distribution along the chromosomes, local co-enrichment analysis was performed. Firstly, an enrichment score (*ES*) was calculated for a gene set at each chromosome band, which was also displayed as the intensity of that region as shown in Fig 2A [32]. Given gene set *X*:*{x*_*1*_,*x*_*2*_,*…x*_*N*_ *}*, chromosome band *B*:*{b*_*1*_,*b*_*2*_,*…b*_*M*_ *}*, the number of genes in each band *G*:*{g*_*1*_,*g*_*2*_,*…g*_*M*_ *}*, then *ES* score was calculated as following.

$$ES\left(X,bi\right)=\frac{Count\left(X\cap genes\in bi\right)}{N\times gi}$$ (2)

Then, a *ES* score vector *EX*:*{ex*_*1*_,*ex*_*2*_,*…ex*_*M*_ *}* was got for gene set *X* across all bands in *B*. Similarly, another vector *EY*:*{ey*_*1*_,*ey*_*2*_,*…ey*_*M*_ *}* could be got for another gene set *Y*. Finally, the similarity (*LCE*) between the two color bands of *X* and *Y* was measured as the Euclidian distance between them.

$$LCE\left(X,Y\right)=1000\times \sqrt{\sum_{i=1}^{M}{\left(exi-eyi\right)}^{2}}$$ (3)

### Global closeness in protein-protein interaction (PPI) network

To evaluate the topological closeness between DEGs and DASGs in the PPI network, average shortest path length between them was used as the measure. Average shortest path length is a concept in network topology that is defined as the average number of steps along the shortest paths for all possible pairs of network nodes, which is a measure of the efficiency of information transport in the network [33]. This measure is also applicable on two-subnetwork issues by calculating the average path distance for all possible pairs of nodes from these two networks, x and y.

$$Dis\;\left(\text{x},\;\text{y}\right)=\frac{\sum_{\text{i}=1}^{\text{i}=\text{M}}\sum_{\text{j}=1}^{\text{j}=\text{N}}\text{dis}\left(\text{i},\;\text{j}\right)}{M\times N}$$ (4)

Where dis(i, j) is the shortest path distance between the *i*-th gene of subset x and the jth gene of subset y. In this analysis, the background network was built using the whole PPI data from online database Human Protein Reference Database (HPRD).

### Functional semantic similarity on Gene Ontology (GO) tree

On the tree of Gene Ontology, genes are categorized into different functional groups named as GO terms based on the biological processes in which they are involved. Based on the graph structure of GO, semantic similarity between any two GO terms was calculated as described in Wang et.al [34] using GOSemSim package [35]. Then the semantic similarity between two gene sets such as DEGs and DASGs could be calculated as the average of all possible pairs of GO terms involving genes in these two gene sets. Given two lists of GO terms, *go*_*1*_ and *go*_*2*_ involving genes in two gene sets, *X* and *Y*, the semantic similarity *SemSim* was defined as:

$$SemSim\left(g1,g2\right)=\frac{\sum_{i=1}^{m}\sum_{j=1}^{n}\sqrt{sim\left(go_{1i},go_{2j)}\right)}}{m\times n}\times \frac{1}{\sqrt{N_{GO}}}$$ (5)

Where *N*_*GO*_ is the total amount of GO terms used for semantic similarity calculation.

## Results

### Summary of RNA-seq results for BEL-7402 cells treated with berberine

The whole transcriptomic profiling of BEL-7402 cells treated with or without berberine was assessed at base-pair resolution via RNA sequencing. After low-quality reads (as mentioned in Read alignment section, Materials and Methods) were cleaned, approximately 30 million reads out of ~41.5 million raw reads (reads retention rate: >70%, GC content: 45~46%, sequence length:36~100 bp) were mapped to reference genome for each sample (Table 1). According to the “*Standards*, *Guidelines and Best Practices for RNA-Seq*” adopted by ENCODE [36], this sequencing depth is good for differential expression analysis and is acceptable for alternative splicing analysis in human transcriptome.

**Table 1:** General Statistics of Reads Alignment Process.

	Ctrl		Berberine	
	Count(left+right)	%	Count(left+right)	%
raw reads	41671690	100.00	41434030	100.00
clean reads	33260886	79.81	32645126	78.78
mapped reads	29934588	71.83	29109042	70.25

### Differentially expressed and alternatively spliced genes induced by berberine treatment

After analyzed by using DESeq software, RNA-seq results returned 343 DEGs in BEL-7402 cells after treated with berberine, with 111 up-regulated and 232 down-regulated (Table A in S1 File). Interestingly, biological process response to bacterium (GO: 0009617) was found to be significantly enriched by up-regulated DEGs (Table 2), which is consistent with the clinical usage of berberine as a broad-spectrum anti-microbial medicine [3]. And we found the genes related to positive regulation of angiogenesis were significantly down-regulated by berberine, which accounts for the anti-angiogenetic activity of berberine reported in previous works. [37–39] According to KEGG pathway enrichment analysis, the DEGs were significantly enriched in p53 signalling pathway (hsa04115) and several cancer-related pathways such as Jak-STAT signalling pathway (hsa04630), Apoptosis (hsa04210) and Pathways in cancer (hsa05200) (Table 3). The Jak-STAT signalling pathway is a major signalling involved in regulation of the immune system, suggesting the immunity-regulating activity of berberine may contribute to its anti-cancer effect.

**Table 2:** Gene Ontology Enrichment Result of DEGs and DASGs.

GeneClass	GO_ID	GO term	Count^#^	p-value
DEG_up	GO:0006334	nucleosome assembly	8	<0.001
DEG_up	GO:0043066	negative regulation of apoptosis	11	<0.001
DEG_up	GO:0009617	response to bacterium	8	<0.001
DEG_up	GO:0045595	regulation of cell differentiation	11	<0.001
DEG_up	GO:0045637	regulation of myeloid cell differentiation	5	<0.001
DEG_up	GO:0002521	leukocyte differentiation	6	<0.001
DEG_up	GO:0051174	regulation of phosphorus metabolic process	10	<0.010
DEG_up	GO:0030097	hemopoiesis	7	<0.010
DEG_up	GO:0051726	regulation of cell cycle	8	<0.010
DEG_up	GO:0031667	response to nutrient levels	6	<0.010
DEG_up	GO:0006986	response to unfolded protein	4	<0.010
DEG_up	GO:0008284	positive regulation of cell proliferation	8	<0.010
DEG_down	GO:0006350	transcription	58	<0.001
DEG_down	GO:0051252	regulation of RNA metabolic process	52	<0.001
DEG_down	GO:0045766	positive regulation of angiogenesis	5	<0.001
DEG_down	GO:0031328	positive regulation of biosynthetic process	18	<0.010
DEG_down	GO:0051174	regulation of phosphorus metabolic process	14	<0.010
DEG_down	GO:0042127	regulation of cell proliferation	19	<0.010
DEG_down	GO:0043067	regulation of programmed cell death	19	<0.010
DEG_down	GO:0007050	cell cycle arrest	6	<0.010
DEG_down	GO:0008284	positive regulation of cell proliferation	12	<0.010
DEG_down	GO:0006915	apoptosis	15	<0.010
DASG	GO:0007049	cell cycle	48	<0.001
DASG	GO:0051276	chromosome organization	35	<0.001
DASG	GO:0065003	macromolecular complex assembly	41	<0.001
DASG	GO:0008104	protein localization	47	<0.001
DASG	GO:0006259	DNA metabolic process	30	<0.001
DASG	GO:0045184	establishment of protein localization	40	<0.001
DASG	GO:0006468	protein amino acid phosphorylation	36	<0.001
DASG	GO:0033554	cellular response to stress	32	<0.001
DASG	GO:0006350	transcription	85	<0.001
DASG	GO:0045926	negative regulation of growth	11	<0.010
DASG	GO:0051640	organelle localization	10	<0.010
DASG	GO:0016044	membrane organization	23	<0.010
DASG	GO:0043407	negative regulation of MAP kinase activity	6	<0.010
DASG	GO:0008219	cell death	34	<0.010
DASG	GO:0008361	regulation of cell size	14	<0.010
DASG	GO:0042770	DNA damage response, signal transduction	8	<0.010

^#^the number of DEGs or DASGs in each Gene Ontology Biological Process term.

**Table 3:** KEGG pathway enrichment result of DEGs and DASGs.

GeneClass	KEGG_ID	KEGG pathway	Count^#^	p-value
DEG	hsa04115	p53 signaling pathway	7	<0.001
DEG	hsa05020	Prion diseases	5	<0.010
DEG	hsa04060	Cytokine-cytokine receptor interaction	12	<0.010
DEG	hsa05322	Systemic lupus erythematosus	7	<0.010
DEG	hsa04640	Hematopoietic cell lineage	6	<0.050
DEG	hsa04630	Jak-STAT signaling pathway	8	<0.050
DEG	hsa04210	Apoptosis	6	<0.050
DEG	hsa04710	Circadian rhythm	3	<0.050
DEG	hsa05200	Pathways in cancer	11	<0.050
DASG	hsa04150	mTOR signaling pathway	6	<0.050
DASG	hsa04144	Endocytosis	12	<0.050
DASG	hsa04810	Regulation of actin cytoskeleton	13	<0.050
DASG	hsa00310	Lysine degradation	5	<0.050
DASG	hsa04360	Axon guidance	9	<0.050

^#^the number of DEGs or DASGs in each pathway.

Meanwhile, alternative splicing analysis was carried out using AltAnalyze software. The results showed that 1083 differentially alternatively spliced exons, corresponding to 867 DASGs (Table B in S1 File) were identified in BEL-7402 cancer cells after berberine treatment. Taken RLIM gene as an example (Fig 1), the inclusion level of exon 2 in the matured mRNA was significantly changed (splicing index < 0.5) after berberine treatment, while there was only a small change in its whole gene expression level. In comparison with RLIM, there was dramatically variation (fold-change > 6) in the gene-expression level of NFKBIA (as an example of DEG) but no significant changes (splicing index ≈ 1) in the inclusion of its exons (Fig 1). As shown in Table 2, the DASGs were involved in a variety of biological processes such as cell cycle (GO: 0007049), chromosome organization (GO: 0051276), macromolecular complex assembly (GO: 0065003) and cellular response to stress (GO: 0033554). Pathway enrichment analysis represented that these DASGs were enriched in PI3K-AKT-mTOR signalling pathway (has: 04150) and regulation of actin cytoskeleton (has: 04810) (Table 3), which all highly related with cell cycle process and cell proliferation [40]. Clearly, these results suggested that these DASGs might also be related with the anticancer action of berberine.

### Statistical analysis of the functional cross-talking between DEGs and DASGs

To explore whether there is any kind of functional cross-talking between DEGs and DASGs, statistical tests were carried out from three primary but different perspectives.

#### Local co-enrichment among chromosome sequences

Intuitively, it should be tested firstly whether those DEGs and DASGs are enriched together at the same or nearby regions on the chromosomes. To carry out this exam, an enrichment score was assigned to each band on all the chromosomes for DASGs and DEGs respectively [32]. As shown in Fig 2A, those DEGs and DASGs exhibited quite different enrichment distributions globally. However, patterns could be found in each local region. Most of the DASG-enriched sites are at or near the sites enriched by DEGs. To quantify the co-enrichment pattern between DEGs and DASGs, Euclidean distance (0.941) was calculated between the enrichment scores on all chromosome bands of DEGs and DASGs. To test the statistical significance of the distance between DEGs and DASGs, simulations by randomly sampling two gene sets in the same size of DEGs and DASGs were carried out for 10000 times to produce the distribution of the Euclidean distance between any two gene sets on the chromosomes. As shown in Fig 2B, the distance between DEGs and DASGs was significantly shorter than those between randomly sampled sets. These results clearly demonstrated that the DEGs and DASGs are physically co-enriched on the corresponding chromosomes.

#### Global closeness based on protein-protein interaction (PPI) network

**Fig 3:**
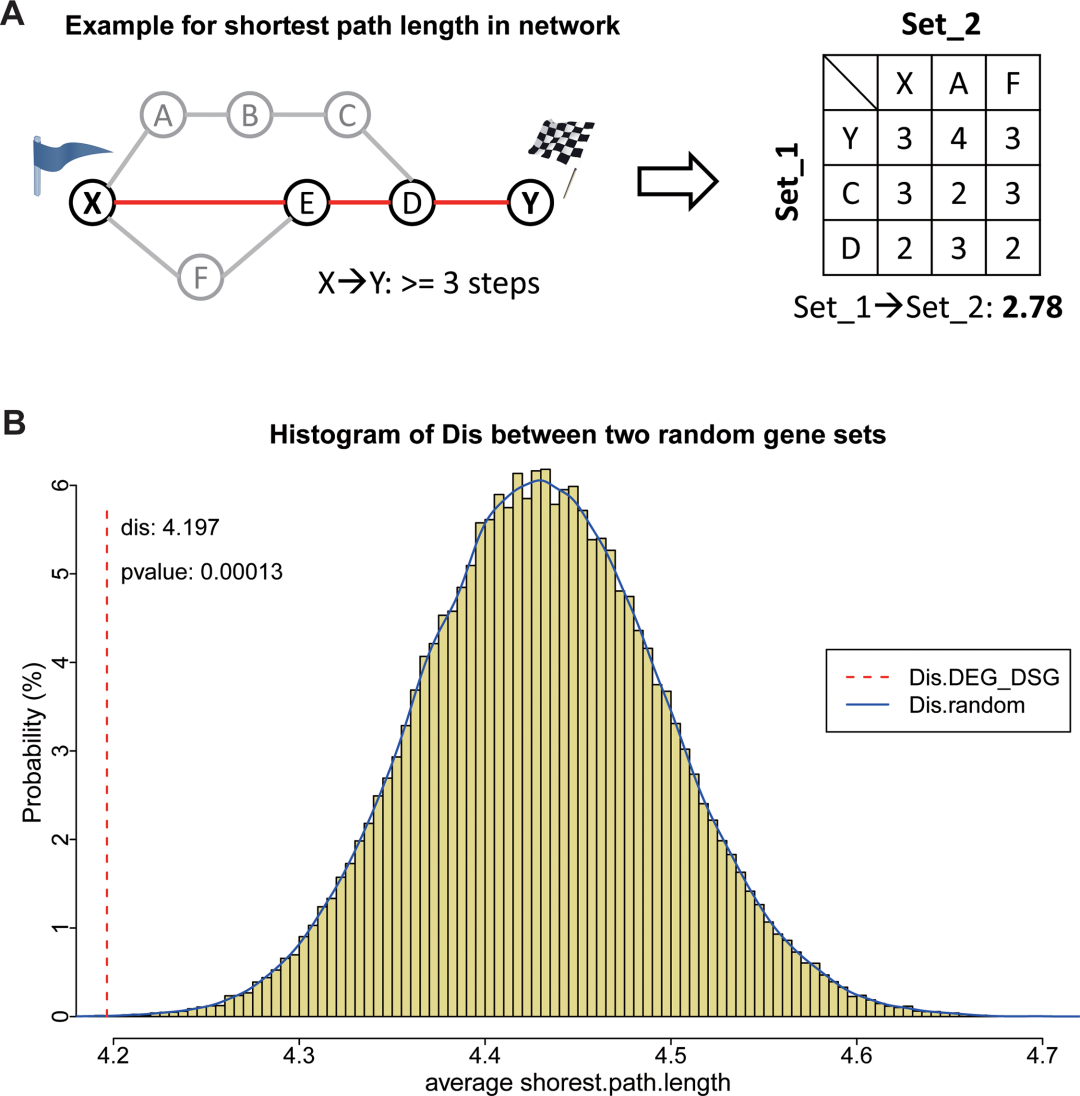
The PPI closeness (averaged shortest path distance) between DEGs and DASGs. A. An example illustration of the closeness between two gene sets. Each circled point represents a node in the network and each line represents the edge between two nodes. The red-colored path is the shortest path between X and Y in this network, which is with 3 steps. The right part is the matrix of shortest path length between all possible pairs from two gene sets (set_1{Y, C, D}, set_2{X, A, F}). And their averaged shortest path length is 2.78. B. The blue curve above the yellow histogram fits the distribution of closeness between two random gene sets in the same sizes of DEGs and DASGs respectively. The red dotted line refers to the distance between DEGs and DASGs. The p-value represents the proportion of gene set pairs with shorter distance than that between DEGs and DASGs.

Following the above result, additional efforts were taken to measure the functional connection between these two gene sets based on PPI network. At first, 100000-time random simulations were performed to build the distribution of averaged shortest path distance between any two gene sets in the same scales as DEGs and DASGs [33]. Then, the averaged distance between DEGs and DASGs were calculated and compared with the distribution. As shown in Fig 3, the former is far away from the mean of the simulated distribution at a statistical significance of p-value < 0.05. The results indicated that these DEGs and DASGs might be closely connected through protein-protein interaction.

#### Functional semantic similarity on Gene Ontology (GO) tree

**Fig 4:**
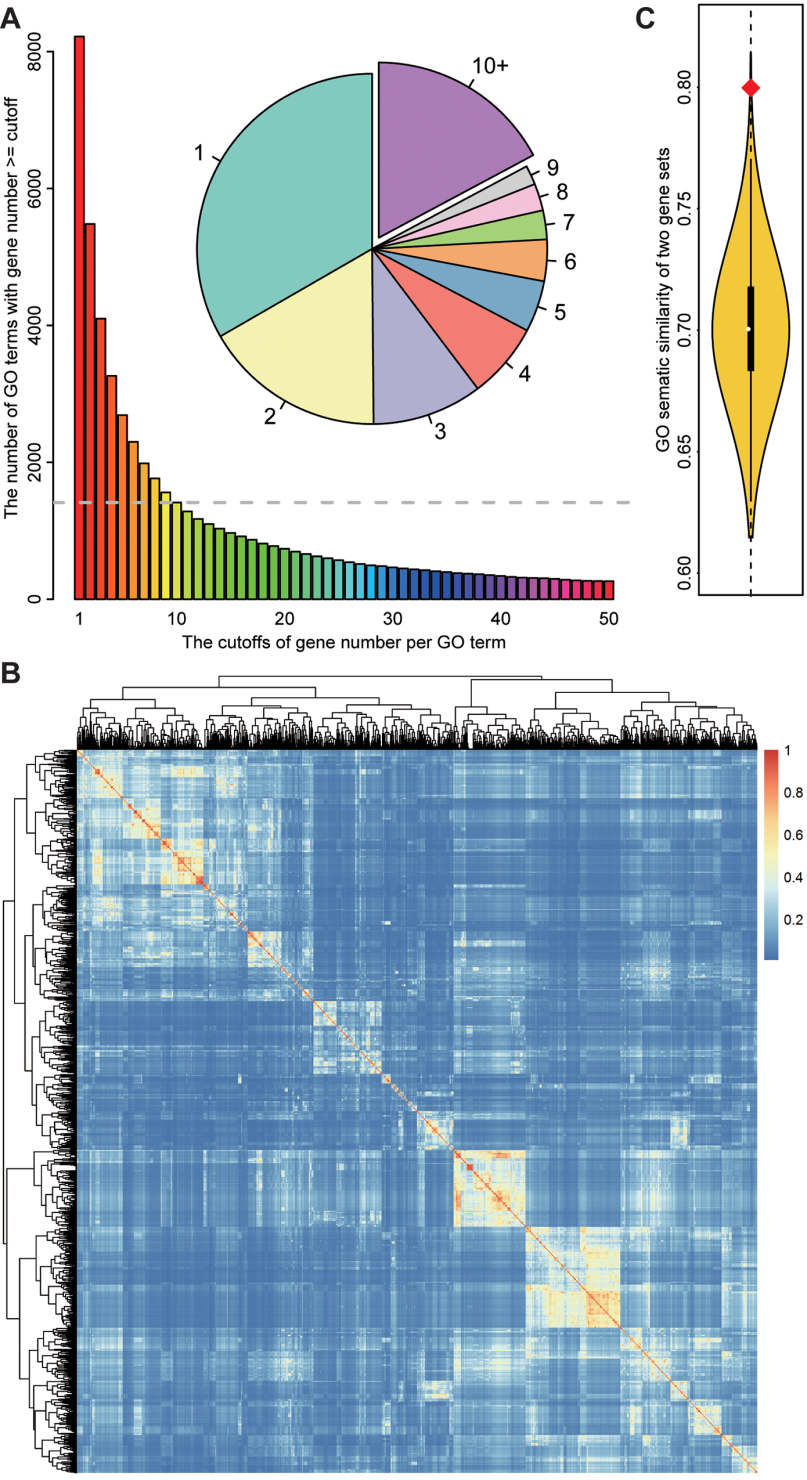
The Gene Ontology semantic similarity between DEGs and DASGs. A. The pie plot shows the proportion of GO terms with the corresponding gene number. The barplot represents the number of GO terms with genes more than each cutoff of gene number. And the part of GO terms with more than 10 genes were selected for further calculation. B. the heatmap of GO semantic similarity between each two selected GO terms. The warmer the color is, the higher the similarity is between the two terms. C. The curve represents the distribution of semantic similarity between two randomly sampled gene sets in the same size of DEGs and DASGs respectively. The red diamond point refers to the semantic similarity between DEGs and DASGs.

While DEGs and DASGs were found to have a significantly shorter distance compared with random-sampled gene sets according to PPI network, this kind of functional correlation is indirect and not with strong biological significance. Thus, a more straightforward method was employed to measure the functional correlation between DEGs and DASGs based on the biological processes affected by them [35]. To balance the computational efficiency and measurement robustness, those GO biological process terms with at least 10 genes were select as the reference biological process set (Fig 4A). A semantic similarity score was assigned to each two GO terms based on the graph structure of GO, as illustrated in Fig 4B. Then an averaged semantic similarity score was calculated over all pairs of GO terms respectively from the biological processes affected by DEGs and DASGs using the best-match average strategy. And the procedures were performed on two randomly sampled gene sets in the same scale as DEGs and DASGs for 10000 times to get the distribution of the averaged semantic similarity. As shown in Fig 4C, the averaged semantic similarity between DEGs and DASGs is extremely higher than two random-pickup sets, indicating that these two set genes might be functionally connected.

## Discussion

Transcription and alternative splicing are two important biological processes during gene expression, which together determine the proteome of cells under a certain condition. In this study, for exploring the functional connection between differentially expressed genes and differentially alternative spliced genes in cancer cells treated with berberine, the mRNA profiling of BEL-7402 cells was collected via RNA-seq. Then, both the DEGs and DASGs induced by berberine were carefully identified by a suite of sequence analysis software. Based on the DEGs functional enrichment analysis, two potential mechanisms underlying the cell cycle arrest property for berberine were suggested as below.

1. **Berberine might induce p53-dependent G1 phase arrest of BEL-7402 cancer cells.** After BEL-7402 cells were treated with berberine, five genes (*BBC3*, *FAS*, *CCNG2*, *GADD45B*, and *IGFBP3*) were up-regulated and two genes (*CASP9*, *THBS1*) were down-regulated (Table C in S1 File) in p53 signaling pathway. Notably, the five up-regulated genes are downstream targets of p53 [41–45]. Meanwhile, p53 was found to be expressed at a modest level in BEL-7402 cells before and after berberine treatment. The wild-type p53 status in BEL-7402 cell line was also confirmed by other researchers [46, 47]. These results suggested that p53 signaling pathway might be influenced under berberine perturbation. As is known, p53 is a crucial cell cycle checkpoint protein that will cause cell cycle arrest responding to DNA damage [48]. Two cell cycle-related genes (CDKN2D, GADD45B), which are in the downstream pathway of p53, were found to be up-regulated after berberine treatment. They could inhibit the activity of CDKs that regulate the G1-S phase transition. And Cell cycle arrest at G1 phase in BEL-7402 cells induced by berberine was also found in our previous work through flow cytometric analysis.[8] Taken together, our results indicated that berberine might inhibit BEL-7402 cell proliferation by inducing G1 cell cycle arrest in a p53-dependent manner. Additionally, p53-dependent cell cycle arrest at G1 phase was also reported in several other cancer cell lines after berberine treatment.[37–39] Our previous studies reported the cell cycle arrest effect of berberine by targeting calmodulin, which might be the upstream signal of p53 pathways [8].
2. **Berberine might suppress the production of inflammatory cytokines through NFκB inhibition.** RNA-seq results indicated that *NFKBIA* (*IκB*) was significantly up-regulated by berberine treatment (Table D in S1 File). As an inhibitor of NFκB, *IκB* can hinder the translocation of NFκB from cytoplasm to nucleus [49]. Previous studies have proved that the inhibition of NFκB may influence the expression of many inflammatory factors which could promote tumor cell growth and facility tumor invasion and metastasis [50]. In this study, five cytokines (*IL-6*, *IL-8*, *IL1A*, *IL-33* and *IL11*) (Table D in S1 File) were found to be down-regulated after berberine treatment according to the RNA-seq results. Notably, all these cytokines have been reported as pro-inflammatory factors. For example, *IL-6* and *IL-8* were reported to be significantly high expressed and play an important role in migration and sustainable proliferation of human liver cancer cells [51–53]. In addition, the silencing of *IL-8* by siRNA could inhibit proliferation and delay the G1 to S cell cycle progression in breast cancer cells or prostate cancer cells lines [54]. This result suggested that suppressing the production of inflammatory cytokines through NFκB inhibition might also contribute to the anti-proliferative effect of berberine in BEL-7402 cells.

Meanwhile, according to the functional annotation on those DASGs, the splicing pattern of a group of cell cycle related genes were also interfered with berberine treatment (Table 2). In addition, the connections between the DEGs and DASGs were measured by statistically analyzing local co-enrichment among chromosomes, network distance and functional semantic similarity on GO trees. The results indicated that these DEGs and DASGs might be connected and functionally cross-talked.

**Fig 5:**
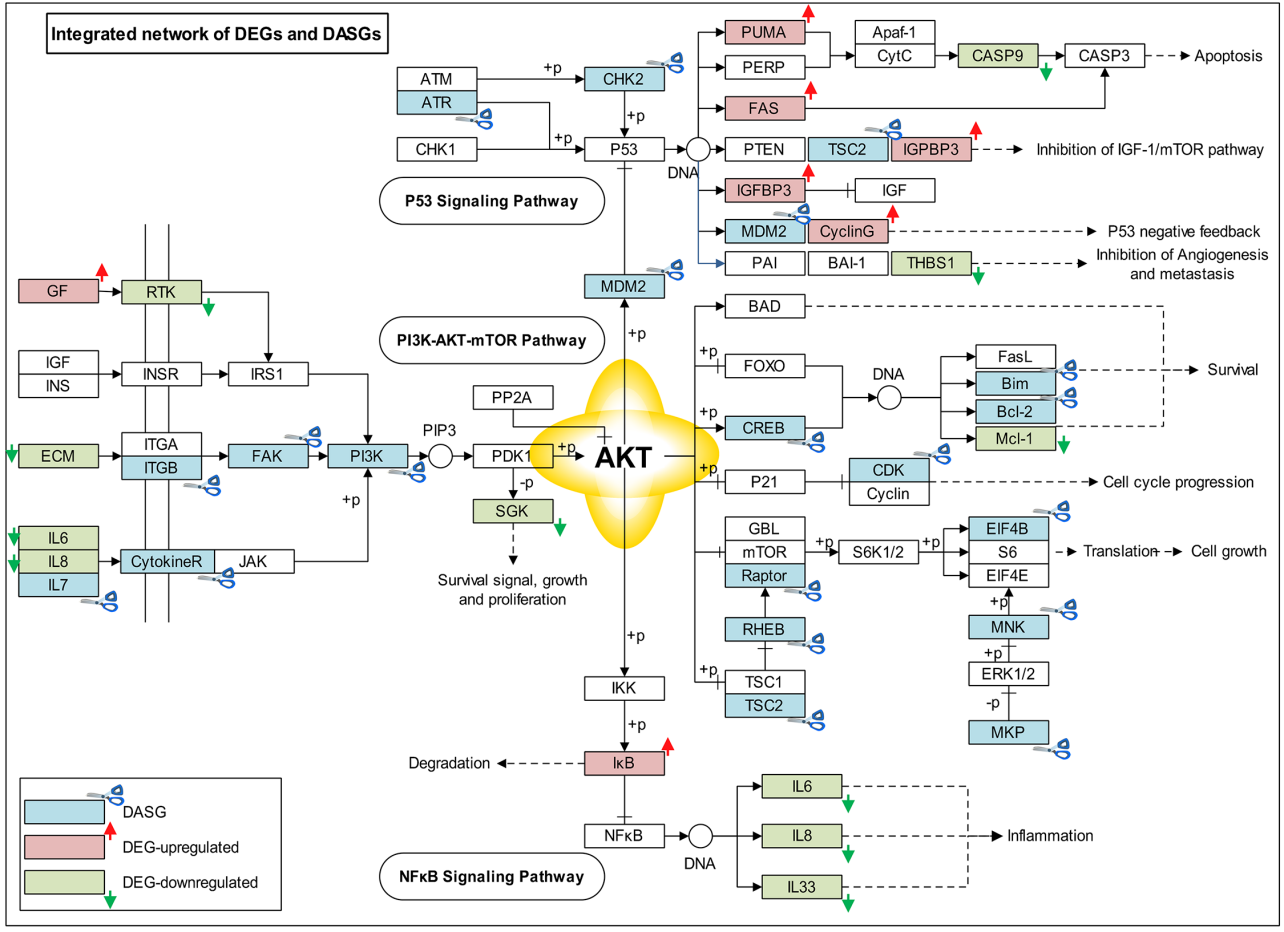
The integrated network affected by DEGs and DASGs. This plot shows an integrated gene-regulation network which is built based on those KEGG pathways affected by DEGs and DASGs. In the plot, red boxes represent those DEGs which are up-regulated; the green ones represent those DEGs which are down-regulated. Those DASGs are displayed as blue-colored boxes.

In addition to overall statistically analysis of the connection between DEGs and DASGs, an integrated network included 15 DEGs and 18 DASGs was re-constructed based on p53 signaling pathway, PI3K-AKT-mTOR pathway, and NFκB signaling pathway (Fig 5). In light of this network, two potential functional connections between DEGs and DASGs were suggested as below.

1. **DEGs and DASGs synergically regulate p53 pathway.** In the upper part in Fig 5, two genes (*CHK2*, *ATR*) were differentially alternative spliced, which are responsible for the phosphorylation and activation of p53 [55, 56]. Meanwhile, another four genes (*PUMA*, *FAS*, *IGF-BP3*, *CyclinG*) were all significantly overexpressed, which are the transcriptional targets of p53 [41–45]. These results highly suggested that these DEGs and DASGs might act synergistically on p53 pathway, thereby inhibit cell cycle progression in cancer cells.
2. **DEGs and DASGs synergically regulate PI3K-AKT-mTOR pathway.** Considering the *PI3K-AKT-mTOR* part in the integrated network (middle part in Fig 5), DASGs were widely distributed both at the upstream and downstream of this pathway. In addition, this pathway seems to play a linking role between p53 pathway and NFκB pathway (Fig 5). For example, *AKT* is involved in the regulation of NFκB activation through the phosphorylation of IκB kinase (*IKK*), which could phosphorylate *IκB* and induce its degradation [57]. Based on DEGs functional analysis, both p53 signalling pathway and NFκB signalling pathway were found to contribute to the anti-proliferative effect of berberine. Moreover, via the DASGs-perturbed PI3K-AKT-mTOR pathway, these two pathways might be linked together for generating berberine’s anticancer effect.

Thus, based on the findings in the current study, we would like to draw attention that in addition to differential expression, differential splicing is very important as well to regulate the cellular activities. The biological outcome of differential splicing is actually the collaborative effects of multiple DASGs coupling with DEGs [13]. Preferably, further experimental validation is desirable when key factors can be identified or the multi-targeting interference could be achieved in one experiment.

## Conclusion

In this study, the genome-wide profiling of transcription and alternative splicing of BEL-7402 cells treated with berberine were analysed by RNA-seq. The results showed that the DEGs and DASGs induced by berberine were functionally enriched in the p53 and cell cycle signalling pathway. Importantly, our results indicated that these two sets of genes might be functionally cross-talked and jointly contribute to the anticancer effect of berberine. This RNA-seq study gave us a better understanding of the molecular mechanisms of anti-cancer effect of berberine. In addition, it has provided new clues for further researches of pharmacological activity of berberine as well as other drugs.

## Supporting Information

S1 File Information of all selected DEGs and DASGs, and those DEGs in specific pathways. There are four supplementary tables. Table A, information about all the DEGs. Table B, information about all the DASGs. Table C, expression data of DEGs in p53 signaling pathway. Table D, expression data of DEGs in NFκB pathway.
(XLSX)

## Abbreviations

DASG: Differentially Alternative Spliced Genes between two samples
DEG: Differentially Expressed Genes between two samples
